# The effects of *Artemisia annua* nutritional supplementation at varying concentrations on broiler growth, economic yield, and gene expression levels of certain antioxidant, inflammatory, and immune genes

**DOI:** 10.14202/vetworld.2024.1318-1327

**Published:** 2024-06-19

**Authors:** Maha Mamdouh, Seham F. Shehata, Amira El-Keredy, Dina A. Awad, Talaat Khedr El-Rayes, Mohamed M. M. Elsokary, Samar H. Baloza

**Affiliations:** 1 Department of Physiology, Faculty of Veterinary Medicine, Benha University, PO 137386, Benha, Egypt; 2 Veterinary Economics and Farm Management, Department of Animal Wealth Development, Faculty of Veterinary Medicine, Benha University, PO 137386, Benha, Egypt; 3 Department of Genetics, Faculty of Agriculture, Tanta University, Tanta, Egypt; 4 Department of Food Hygiene and Control, Faculty of Veterinary Medicine, Benha University, PO 13736, Benha, Egypt; 5 Department of Animal Production, Faculty of Agriculture, Tanta University, Tanta, Egypt; 6 Veterinary Medicine and Food Security Research Group, Faculty of Health Sciences, Higher Colleges of Technology, Abu Dhabi 17155, United Arab Emirates; 7 Department of Theriogenology, Faculty of Veterinary Medicine, Benha University, PO 13786, Benha, Egypt; 8 Genetics and Genetic Engineering, Department of Animal Wealth Development, Faculty of Veterinary Medicine, Benha University, PO 137386, Benha, Egypt

**Keywords:** *Artemisia annua*, broiler, carcass traits, economics, immunity, mRNA gene expression

## Abstract

**Background and Aim:**

*Artemisia annua* (AA), used as a growth promoter in poultry, lowers feed costs and enhances economic efficiency. This study aimed to assess the impact of varying AA concentrations on broiler chicken growth, gene expression, and profitability.

**Materials and Methods:**

Two hundred 1-day-old male Cobb chicks were randomly allocated into four treatment groups, each containing five replicates and 10 birds. The experimental groups consisted of G1 (basal diet), G2 (basal diet with 0.3% AA), G3 (basal diet with 0.6% AA), and G4 (basal diet with 0.9% AA). The birds had continuous access to feed and water throughout the study. The experiment lasted for 42 days. We measured the growth performance (Feed intake, Life weight), carcass traits (weight after slaughter, dressed carcass, heart, gizzard, spleen, giblet and thymus weight), liver and spleen antioxidants (CAT, GSH, SOD), and gene expression of anti-inflammatory and immune- related genes.

**Results:**

The primary findings revealed that the addition of 0.6% AA had a positive impact (p < 0.05) on all investigated variables compared with the control and other groups. Dietary supplementation with 0.6% AA led to increased breast, giblet, skeleton, and total yield, and net return compared with the control group. Supplementation with AA exhibited antioxidant, anti-inflammatory, and immunological effects through improved levels of antioxidant superoxide dismutase (SOD), catalase (CAT), and glutathione peroxidase (GSH-Px) in tissue homogenates of the liver and spleen. It also upregulated the relative messenger RNA levels of anti-inflammatory interleukin (IL)-10, SOD, CAT, and GSH-Px, whereas IL-1β and tumor necrosis factor-alpha were downregulated.

**Conclusion:**

The study found that AA is a promising replacement for antibiotics in poultry farming as a growth promoter for chickens. 0.6% AA in the broiler diet yielded the best results, striking a balance between superior performance and robust economic benefits.

## Introduction

*Artemisia annua* (AA) is a promising feed additive for increasing live weight in broiler chickens. Studies show that AA supplementation in broiler chickens, due to the presence of artemisinin and flavonoids, can enhance nutrient absorption, digestion, and metabolism, resulting in increased weight gain [1, 2]. These compounds possess anticancer and antimicrobial properties against antibiotic-resistant pathogens and stimulate the immune system. AA’s antioxidant properties, including free radical scavenging, emerge from its richness in antioxidant compounds such as phenolics and flavonoid complexes. These antioxidant molecules work together to neutralize harmful free radicals [3, 4].

By 2050, poultry meat will surpass other meats in consumption, with demand doubling current levels. Poultry plays a crucial role and is a vital source of high-quality protein and essential nutrients for underprivileged populations in developing countries, stretching beyond affordability [5, 6]. The poultry industry is shifting toward incorporating antibiotic substitutes, such as phytogenic feed additives, in broiler feed to modulate chicken gut microorganisms, enhance productivity, and positively impact feed intake (FI) and digestion efficiency [7]. Additives significantly enhance feed efficiency, growth, and disease resistance by balancing intestinal microbiota, fortifying intestinal integrity, and hindering pathogens, resulting in an optimized immune system. Assessing the herb’s impact on the immune system and its economic worth is crucial for a complete appraisal of its benefits [8]. The antimicrobial and antioxidant properties of AA enhance the health and well-being of broiler chickens. For maximum benefits and immune response enhancement with minimal adverse effects, the optimal dosage, duration, and formulation of AA supplementation should be carefully considered [6, 9, 10].

The antioxidative and anti-inflammatory properties of AA in animals lead to improved intestinal configurations in heat-stressed broilers, enhancing their immunity and antioxidant capacity [3, 11]. Overproduction of reactive oxygen species (ROS) can cause extensive damage to DNA, proteins, and lipids. Cellular antioxidant defense systems, including superoxide dismutase (SOD), glutathione peroxidase GSH-Px catalase (CAT), and reduced glutathione (GSH), have evolved to protect cellular components against ROS-induced damage [12].

Wan *et al*. [11] reported enhanced activities of CAT and SOD in serum and liver under thermoneutral conditions due to AA supplementation in broiler diets. The antioxidant capacity of broilers is enhanced by AA’s phenolic and flavonoid content [13]. A decrease in malondialdehyde (MDA) levels in both serum and liver samples indicates enhanced protection against oxidative stress. 1 g/kg AA in the diet improved immune and antioxidant functions of heat-stressed broilers. Antioxidant enzymes such as SOD, CAT, and GSH-Px in the liver were enhanced while MDA accumulation was reduced, indicating a decrease in oxidative stress [14, 15]. Carefully considering the optimal dosage, duration, and formulation of AA supplementation is crucial for maximizing benefits while minimizing adverse effects.

This study investigated the impact of different AA concentrations (0.3%, 0.6%, and 0.9%) on broiler growth performance and gene expression. In addition, we focused on the primary antioxidant enzyme system in hepatic tissue, including SOD, glutathione peroxidase (GPX), and CAT, synergistic with the action of some immune-related genes in splenic tissue and interleukin (IL)-1β, tumor necrosis factor-α (TNF-α), and IL-10 in broiler chicken.

## Materials and Methods

### Ethical approval

All procedures were performed after obtaining ethical approval from the Animals Care and Use Committee Research Ethics Board, Faculty of Veterinary Medicine, Benha University, under ethical number BUFVTM-28-10-22.

### Study period and location

The study was conducted from September 9 to October 21, 2022. The study was conducted at the Center of Experimental Animal Research, located within the Faculty of Veterinary Medicine at Benha University, Egypt.

### Birds and their diets

The study used two hundred 1-day-old male Cobb-500 broiler chicks from local hatcheries. All chick populations were maintained under identical hygienic and managerial conditions during the study. The chicks went through transportation, individual weighing, wing banding and were housed in well-ventilated litter floor pens. The chicks were kept in their pens. Each had dimensions of 50 cm × 100 cm × 100 cm. 23 h of light and 1 h of darkness were continuously rotated throughout the study to mimic natural daylight patterns.

During the 6-week experiment, the nutritional needs of growing chicks were met through a personalized feeding regimen. The diet followed the Cobb-500 broilers management guide’s nutritional guidelines, using corn-soybean meal basal formulations (Accessible at: https://www.cobb-vantress.com/assets/5a88f2e793/Broiler-Performance). During the 6-week experiment, the chicks were fed pelleted diets tailored to their age. During the first 2 weeks, chicks were given a starter diet. From the 3^rd^ to 6^th^ week, they received a grower and finisher diet. The chemical compositions of each basal diet’s components are listed in Table-1.

**Table-1:** Ingredients and the calculated chemical composition of the basal diets used during different growth stages of broiler birds.

Items	Starter diet	Grower diet	Finisher diet
Yellow corn	54.67	58.28	62.62
Soybean meal, 46%	36	33.8	28.9
Vegetable oil	2.5	3.5	4.5
Corn gluten meal, 60%	2	0	0
Di calcium phosphate	1.7	1.45	1.33
Limestone	1.45	1.35	1.2
L-Lysine	0.33	0.29	0.23
Sodium chloride	0.32	0.3	0.3
Vitamin and mineral premix^1^	0.3	0.3	0.3
DL-Methionine	0.28	0.27	0.23
Sodium bicarbonate	0.19	0.17	0.17
Anti-coccidian	0.05	0.05	0.05
Anti-mycotoxin	0.05	0.05	0.05
Anti-clostridia	0.03	0.03	0.03
L-Threonine	0.03	0.04	0
Energy enzyme	0.02	0.04	0.01
Lysomax	0.01	0.01	0.01
Phytase enzyme	0.01	0.01	0.01
Protease B	0.01	0.01	0.01
Choline chloride	0.05	0.05	0.05
Calculated composition			
Crude protein%	23.02	21.03	19.03
MEn kcal/kg	3053.85	3152.05	3224.10
Crude fiber%	2.27	2.25	3.13
Lysine %	1.35	1.25	1.09
Methionine%	0.63	0.59	0.54
Methionine+cysteine%	1.02	0.95	0.86
Threonine %	0.94	0.88	0.77
Calcium %	1.05	0.95	0.85
Available phosphorus%	0.50	0.45	0.42

^1^Each 3 kg contained: Vit. A 12,000,000 IU, Vit. D3 2,000,000 IU, Vit. E 10,000 mg, Vit. K3 2000 mg, Vit. B 11,000 mg, Vit. B2 5000 mg, Vit. B6 1500 mg, Vit. B12 10 mg, Biotin 50 mg, Pantothenic acid 10,000 mg, Nicotinic acid 30,000 mg, Folic acid 1000 mg, Manganese 60,000 mg, Zinc 50,000 mg, Iron 30,000 mg, Copper 10,000 mg, Iodine 1000 mg, Selenium 100 mg, Cobalt 100 mg, carrier (CaCo_3_) added to make the total 3 kg, MEn=Metabolizable energy

### Preparation of AA powder

In June, leaves from undamaged AA trees with consistent green color were harvested from a single location to ensure uniform soil micronutrient levels. Tree was identified by Talaat Khedr El-Rayes (author). During the day, the leaves were turned regularly as they were air-dried to inhibit fungal growth. After 5 days of drying, the leaves were ground into a fine powder using a 0.15-mm sieve. The leaf meal was stored in airtight high-density polyethylene bags at room temperature until use.

11.4% moisture content was identified in the AA’s chemical makeup. 8.3% of the primary macromolecules consisted of carbohydrates, while 24.37 mg/100 g were proteins. The sample comprised 14.2% dietary fiber and 7.5% ash. 6.07% was the moderate fat content. Phytate (140.4 mg/100 g), total tannins (0.61 mg/100 g), and tocopherol (2.74 mg/100 g) were all identified in the analysis.

### Experimental design

Two hundred of broiler chicks were distributed randomly into four groups, each containing five replicate pens. Ten birds per pen received the basal diet (G1), while dietary supplements of 0.3% AA (G2), 0.6% AA (G3), and 0.9% AA (G4) were added to the respective groups’ food.

### Assessment of growth indices

Body weight was determined following the methodology outlined by Shehata *et al*. [16]. FI was determined by subtracting the weight of the feed refused from the initially offered feed weight [17, 18].

### Evaluation of carcass traits

In the 6^th^ week of rearing, 10 birds were randomly chosen from each group. Birds were weighed alive first before being slaughtered with a knife. After bleeding, the birds were then defeathered, including the removal of their heads and legs. The weights of different carcass components, such as the dressed carcass, heart, gizzard, spleen, liver, thymus, and intestine, were measured after manual evisceration. The combined weight of the heart, liver, spleen, gizzard, and thymus (giblets) was computed according to Azam *et al*.’s methodology [19]. The weight (g) of the dressed carcass, excluding giblets and the neck, was recorded 2 h after refrigeration. The weights (g) for the wings, leg quarters, breasts, and frame were recorded after the carcasses were deboned. According to Park *et al*. [20], the collective measurement of chicken parts, including wings, leg quarters, breasts, frame, head, and legs is referred to as chicken skeletons.

### Assessment of SOD, CAT, and GSH levels in liver and spleen homogenates

The supernatant was removed from the homogenate following the manufacturer’s instructions (Sigma-Aldrich, USA). 0.1 g tissue samples were homogenized in 10% solution and centrifuged at 1000× *g*, 4°C, for 10 min. Using bio-diagnostic commercial kits (Sigma-Aldrich), we measured quantitatively the levels of CAT, GSH, and SOD in both spleen and liver homogenates. Each kit’s calorimetric measurements were carefully followed.

### Polymerase chain reaction (PCR)-based quantitation of messenger RNA (mRNA) transcripts

In triplicate reverse transcription PCR (RT-PCR) experiments, gene expression levels of SOD, CAT, and glutathione peroxidase (GPX) were measured in liver samples from each experimental group. Triplicate splenic tissue samples from each experimental group underwent total RNA extraction using TRIzol Reagent (Thermo Fisher Scientific, USA), following the manufacturer’s directions. The RNA was converted to cDNA using Applied Biosystems kits (USA) and then analyzed by quantitative PCR (qPCR) in the 7500 Fast system with SYBR Green master mix (Thermo Fisher Scientific). The qPCR assay measured the amounts of mRNA for IL-1β, TNF-α, and IL-10 cytokines. In the 20 μL reaction, the programmed sequence consisted of an initial stage of 95°C for 30 s, followed by 40 cycles each with steps at 95°C for 5 s, 60°C for 30 s, and 72°C for 20 s. The 5´-3´ primer sequences for qRT-PCR analysis are given in Table-2 [21–25]. Transformed gene expression levels were calculated by the 2^-∆∆Ct^ formula using normalization to β-actin, as reported by Livak and Schmittgen [26].

**Table-2:** Primer sequences (5´-3´) of candidate genes in qReal time PCR.

Gene	Primer (5-3) oligonucleotides	Accession No.	Reference
*SOD*	F: CGGGCCAGTAAAGGTTACTGGAA R: TGTTGTCTCCAAATTCATGCACATG	NM_205064.1	[21]
*CAT*	F: ACTGGTGCTGGCAACCC R: ACGTGGCCCAACTGTCAT	NM_001031215	
*GPX*	F: CAAAGTTGCGGTCAGTGGA R: AGAGTCCCAGGCCTTTACTACTTTC	NM_001163245.1	[22]
*IL 1B*	F: TGCCTGCAGAAGAAGCCTCG R: GACGGGCTCAAAAACCTCCT	NM_204524.1	[23]
*TNF-α*	F: CGCTCAGAACGACGTCAA R: GTCGTCCACACCAACGAG	MF000729.1	[24]
*IL10*	F: CAGACCAGCACCAGTCATCA R: TCCCGTTCTCATCCATCTTCTC	NM_001004414.2	[23]
*β-actin*	F: ACCTGAGCGCAAGTACTCTGTCT R: CATCGTACTCCTGCTTGCTGAT	NM_205518.1	[25]

PCR=Polymerase chain reaction, SOD=Superoxide dismutase, CAT=Catalase, IL=Interleukin, TNF-α=Tumor necrosis factor-α

### Evaluation of economic efficiency

To assess economic efficiency, a comprehensive evaluation of production costs and revenue was conducted. The total production cost (TC) consists of both variable and fixed expenses. The expenses related to feed consumption, veterinary services, labor, chick purchase, utilities, and litter, as described by Al-Khalaifah *et al*. [27], were classified as total variable costs (TVCs). Instead, rent for buildings and equipment made up the total fixed costs. The total feed cost was determined by multiplying the total FI per bird by the cost per kilogram [28, 29]. The total revenue (TR) calculation included breast meat, leg quarters, giblets, skeletons, and litter returns. Net profit (NP) was determined as the difference between TR and TCs, according to Mohammed *et al*. [30].

This study assessed economic efficiency using the benefit–cost ratio (BCR) (TR/TC, TR/TVC, NP/TC, and NP/TVC) as per method described by Shehata *et al*. [29].

### Statistical analysis

Statistical analysis of the results was performed using IBM Statistical Package for the Social Sciences Version 22.0 (IBM Corp., NY, USA) [31]. A one-way analysis of variance was used to establish differences between the treatment groups. To identify the effects of varying levels of AA on different independent variables, contrast analysis was carried out between the control group, AA 0.3%, AA 0.6%, and AA 0.9% experimental groups. At a 5% significance level, Tukey’s test was utilized to identify disparities among the means of the experimental groups. The results are presented as the mean ± pooled standard error.

## Results

### Growth performance and carcass traits

The data in Table-3 indicate the effects of introducing various degrees of AA into broiler diets on factors such as FI, live weight, carcass attributes, and internal organ development throughout the experiment. Including AA in broiler diets led to significantly heavier spleen and giblets (p < 0.05). Birds given AA supplementation showed greater spleen and giblet weights without significant differences in FI, final live weight, or carcass cuts.

**Table-3:** Effect of diet supplemented with *Artemisia annua* (%^−1^ diet) on life weight, some carcass traits and some internal organs of broiler chickens (Mean±standard error).

Item	Control	*Artemisia annua* (%^−1^ diet)			Pooled SEM	p-value			
	
		0.3	0.6	0.9		T	C versus 3% T	C versus 6% T	C versus 9% T
Feed intake	3404.17	3461.17	3485.17	3463.33	20.79	NS	0.77	0.53	0.75
Life weight	2159.74	2161.50	2161.67	2163.00	30.92	NS	1	1	1
Weight after slaughter	2106.67	2115.83	2123.33	2109.17	3.65	NS	1	0.99	0.99
Weight after defeathering	53.33	58.33	58.33	81.67	3.65	NS	0.96	0.96	0.06
Weight after leg and head	1950.00	1945.83	1967.50	1925.00	28.801	NS	1	0.99	0.99
Dressed carcass	1386.05	1388.58	1414.38	1390.73	28.52	NS	0.97	0.72	0.75
Heart weight	9.67	9.5	10	9.5	0.13	NS	0.97	0.81	0.97
Liver weight	50.17	53.83	57.33	57.33	0.75	NS	0.34	0.02	0.02
Gizzard before	74.17	75.00	76.67	79.17	2.10	NS	1	0.97	0.84
Gizzard after	54.67	57.5	62.5	58.83	1.35	NS	0.88	0.21	0.70
Spleen weight	3.33^b^	4.13^a^	3.80^ab^	3.95^a^	0.11	*	0.07	0.43	0.21
Bursa weight	1.80	2.17	2.03	2.10	0.06	NS	0.13	0.47	0.26
Thymus weight	2.65	2.62	2.45	2.55	0.06	NS	1.00	0.62	0.93
Giblet weight	122.28^b^	129.75^b^	138.12^ab^	134.27^a^	1.87	*	0.51	0.03	0.14
Intestine weight	96.67	86.67	80.00	75.00	2.91	NS	0.63	0.21	0.07
Breast without bone	486.33	515.00	508.33	506.67	12.89	NS	0.59	0.70	072
Leg quarters	601.67	613.33	606.67	591.67	15.84	NS	0.99	0.99	0.99
All skeleton weight	393.33	383.33	405.83	379.17	13.33	NS	0.79	0.74	0.71

^1^Means within a row with different superscripts (a, b) significantly differ (*p < 0.05), NS=Not significant, BW=Body weight, SEM=Standard error of the mean, C=Control, and T=Treatment

### Metabolism and gene expression

**Figure-1:**
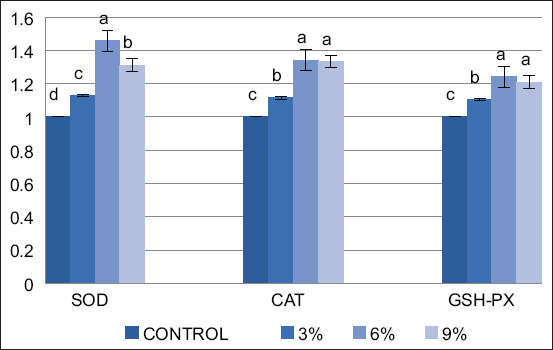
Effect of *Artemisia annua* on the expression of antioxidant-related genes in the liver of broilers, SOD=Total superoxide dismutase, CAT=Catalase, GSH-Px=glutathione peroxidase at 42 days of age with concentration, 3, 6 and 9% *A. annua* in diet. Each value is exhibited as the mean and the standard error of mean (n = 3), different superscript letters (a, b, and c) point to significant differences among experimental groups (p < 0.01), while the same letter means no significant difference among groups.

**Figure-2:**
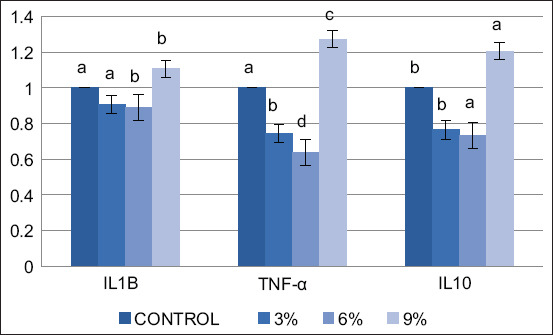
Effect of *Artemisia annua* on the expression of anti-inflammatory and immune-related genes, interleukin 1β, tumor necrosis factor-α, and interleukin 10 in the spleen of broilers at 42 days of age with concentration, 3, 6, and 9% *A. annua* in diet. Each value is displayed as the mean and the standard error of mean (n = 3), different superscript letters (a, b, c, and d) reveal significant differences between experimental groups (p < 0.01), while the same letter means no significant difference among groups.

The data presented in Tables-4 and 5 reveal a significant impact (p < 0.05) of AA supplementation on antioxidant enzyme levels in the liver and spleen tissues of broiler chickens. In the liver, a dose-dependent response was observed, with the highest AA concentrations (0.6% and 0.9%) leading to significantly (p < 0.05) elevated levels of CAT, GSH, and SOD compared with the control and 0.3% AA-supplemented groups. Notably, a similar pattern was observed in the spleen, with all AA-supplemented groups (0.3%, 0.6%, 0.9%) exhibiting significantly (p < 0.05) higher levels of CAT, GSH, and SOD than the control. The expression of liver antioxidant-related genes, the antioxidant activity resulting from dietary AA supplementation is evident in the improved activity of antioxidant enzymes. This enhancement is reflected in substantially higher gene expression levels of CAT, SOD, and GPx with increasing supplementation doses, particularly evident in the 0.6% AA supplemented group (Figure-1). In the 0.3%, 0.6%, and 0.9% AA-supplemented groups, both a linear decrease in pro-inflammatory IL-1β and TNF-α expression (p < 0.01) and an increase in anti-inflammatory IL-10 expression compared with the control group were observed (Figure-2).

**Table-4:** Effect of diet supplemented with *Artemisia annua* (%^−1^ diet) on liver antioxidant of broiler chickens (mean±standard error).

Item	Control	*Artemisia* *annua* (%^−1^ diet)			Pooled SEM	p-value			
	
		0.3	0.6	0.9		T	C versus 3% T	C versus 6% T	C versus 9% T
Liver antioxidant									
CAT (U/g)	1030.25^c^	1252.25^c^	1355^b^	1626.00^a^	***	>0.0001	0.000	0.000	0.000
GSH (mg/g)	46.52^c^	55.98^c^	64^b^	83.00^a^	***	>0.0001	0.000	0.000	0.000
SOD (U/mg)	745.07^c^	752.25^b^	933.04^a^	933.5^a^	***	>0.0001	0.000	0.000	0.000

^1^Means within a row with different superscripts (a, b, c) significantly differ (*p < 0.05, **p < 0.01,

*** p < 0.001), NS=Not significant, CAT=Catalase, GSH=Glutathione, SOD=Superoxide dismutase, SEM=Standard error of the mean, C=Control, and T=Treatment

**Table-5:** Effect of diet supplemented with *Artemisia annua* (%^−1^ diet) on spleen antioxidant of broiler chickens (mean±standard error).

Item	Control	*Artemisia* *annua* (%^−1^ diet)			Pooled SEM	p-value			
	
		0.3	0.6	0.9		T	C versus 3% T	C versus 6% T	C versus 9% T
Spleen antioxidant									
CAT (U/g)	368.25^c^	378.66^b^	378.66^b^	383.25^a^	***	>0.0001	0.000	0.000	0.000
GSH (mg/g)	55.57^c^	64.4475^c^	72.25^b^	88.50^a^	***	>0.0001	0.000	0.000	0.000
SOD (U/mg)	745.07^c^	752.25^b^	933.04^a^	933.5^a^	***	>0.0001	0.000	0.000	0.000

^1^Means within a row with different superscripts (a, b, c) significantly differ (*p < 0.05, **p < 0.01,

*** p < 0.001), NS=Not significant, SOD=Superoxide dismutase, CAT=Catalase, GSH=Glutathione, SEM=Standard error of the mean, C=Control, and T=Treatment

### Economic efficiency

Economic parameters and efficiency indices varied during the experimental period based on the different levels of AA are presented in Table-6. The control and AA-supplemented groups had similar TVCs, TCs, TR, and net returns at the three doses (0.3%, 0.6%, 0.9%). In the 0.6% and 0.3% AA-supplemented groups, TR and NR values were higher. The 0.6% AA group showed significantly higher values for TR/TVC, BCR, NP/TC, and NP/TVC compared to the other groups (0.3%, 0.9%, and control). In contrast, lower sales of broiler cuts and TR were seen in the control group, with the 0.9%, 0.3%, and 0.6% AA-supplemented groups exhibited less decline.

**Table-6:** Effect of diet supplemented with *Artemisia annua* (%^−1^ diet) on the economic parameters and economic efficiency measures during the experimental period of broiler chickens.

Item	Control	*Artemisia* *annua* (%^-1^ diet)			Pooled SEM	p-value			
	
		0.3	0.6	0.9		T	C versus 3% T	C versus 6% T	C versus 9% T
Chick price	6.5	6.5	6.5	6.5	0.00	–	–	–	–
Drug cost	1.4	1.4	1.4	1.4	0.00	–	–	–	–
Vaccine cost	1.1	1.1	1.1	1.1	0.00	–	–	–	–
Disinfectant cost	0.3	0.3	0.3	0.3	0.00	–	–	–	–
TVM	2.8	2.8	2.8	2.8	0.00	–	–	–	–
Water and Electricity	0.15	0.15	0.15	0.15	0.00	–	–	–	–
Equipment	0.15	0.15	0.15	0.15	0.00	–	–	–	–
Labor	1.6	1.6	1.6	1.6	0.00	–	–	–	–
Litter cost	1.35	1.35	1.35	1.35	0.00	–	–	–	–
Building	2.1	2.1	2.1	2.1	0.00	–	–	–	–
TFC	2.25	2.25	2.25	2.25	0.00	–	–	–	–
Feed intake	3404.17	3461.17	3485.17	3463.33	20.79	NS	0.77	0.53	0.75
Feed cost	29.14	29.65	29.86	29.69	0.18	NS	0.75	0.50	0.70
TVC	41.54	42.05	42.26	42.09	0.17	NS	0.75	0.50	0.70
Total cost	43.79	44.30	44.51	44.34	0.17	NS	0.75	0.50	0.70
Litter return	0.55	0.55	0.55	0.55	0.00	–	–	–	–
Breast return	47.78	49.56	51.45	49.35	0.95	NS	0.51	0.19	0.56
Leg quarters return	18.05	18.4	18.2	17.75	0.47	NS	0.79	0.91	0.82
Giblet return	3.67	3.89	4.14	4.03	0.05	NS	0.16	0.16	0.16
Skeleton return	3.93	3.83	4.06	3.79	0.13	NS	0.79	0.74	0.71
Return broiler cuts	73.43	75.69	77.85	74.92	1.40	NS	0.57	0.28	0.71
TR	73.98	76.23	78.40	75.47	1.40	NS	0.58	0.28	0.71
NR	30.18	31.93	33.89	31.13	1.41	NS	0.66	0.61	0.82
Economic efficiency measures									
TR-TC (BCR)	1.69	1.72	1.76	1.70	0.03	NS	0.75	0.45	0.90
TR/TVC	1.78	1.81	1.86	1.79	0.04	NS	0.76	0.47	0.91
NR/TC	0.69	0.72	0.76	0.70	0.03	NS	0.75	0.45	0.90
NR/TVC	0.69	0.72	0.76	0.70	0.03	NS	0.76	0.47	0.90

TVM=Total veterinary management, TFC=Total feed cost, TVC=Total variable cost, TC=Total costs, TR=Total return, NR=Net return, kg=Kilogram, BCR=Benefit cost ratio, SEM=Standard error of the mean, C=Control, and T=Treatment

## Discussion

In poultry feeding, prioritizing gut health to achieve optimal feed conversion ratio (FCR) and growth performance is crucial, especially in the absence of growth-promoting antibiotics. Incorporating various additives into poultry feed results in improved weight gains and a lower FCR for each bird.

We examined the influence of varying AA amounts on final weight, FI, and carcass yield in broiler chickens. According to Wan *et al*. [11] and Panaite *et al*. [8], AA had a minimal impact on broiler performance. While FI, final live weight, and carcass cuts showed no significant differences, the spleen and giblet weights increased remarkably in the AA-supplemented groups. The documented enhancements in spleen and giblet weights in broilers supplemented with AA extract [32] correspond to our findings. Saracila *et al*. [33] did not find significant differences in carcass cuts between AA-supplemented and control groups. The varied results of AA supplementation on FI, live weight, and carcass traits among studies can be explained by differences in AA dosage and duration and the basal diet’s composition. Environmental factors and management practices significantly affect the observed outcomes, as suggested by Saracila *et al*. [34]. Our study found no significant difference in dressed carcass weight, internal organs, breast, leg quarters, and overall skeleton weights when broiler diets were supplemented with varying levels of AA. A substantial rise in spleen and giblet weights (p ≤ 0.05) occurred, in line with Al-Shuwaili *et al*.’s findings [35]. These findings concur with those of Wan *et al*. [11] and Park and Kim [36], who demonstrated that oil extracts could exert stimulatory effects on the digestive system of poultry, enhance liver function, and enhance the activity of pancreatic digestive enzymes.

Oxidative stress in poultry, caused by moldy feed, an inadequate feeding environment, and an imbalance in intestinal flora [37], negatively impact the quality of broiler meat. Recognizing the potential harmful effects of oxidative stress on broiler meat, it is imperative to incorporate dietary antioxidants to shield poultry meat from the damage inflicted by free radicals, especially given its high polyunsaturated fatty acid content [38]. Antioxidant enzymes such as SOD, GSH-Px, and CAT work together in poultry to preserve optimal redox balance. This equilibrium significantly influences the diverse processes of cell signaling, gene expression, stress modulation, and homeostasis preservation [39]. Antioxidant defense’s first line is formed by SOD, a metalloenzyme made up of proteins and metal cofactors. It catalyzes the conversion of harmful superoxide radicals into less damaging oxygen and hydrogen peroxide. This hydrogen peroxide is further neutralized by the combined efforts of GSH-Px and CAT, ultimately breaking it down into harmless water and oxygen [40]. The levels of antioxidant enzymes (SOD, GSH-Px, and CAT) in the liver and spleen supernatants were measured to evaluate the antioxidant power of AA. The control group had significantly (p < 0.05) lower levels of CAT, GSH, and SOD than the AA-supplemented groups. AA supplementation at 0.6% and 0.9% doses significantly (p < 0.05) increased target antioxidant enzyme levels compared to the control group. The liver gene expression data confirmed a significant, dose-dependent increase in SOD, CAT, and glutathione peroxidase levels by day 42 (p < 0.01). Cherian *et al*. [41] and Gholamrezaie [42] confirmed the notable antioxidant and immune-enhancing effects of adding AA to broiler diets. In addition, previous studies by Guo *et al*. [43] and Shi *et al*. [2] demonstrated increased antioxidant enzyme activity in various body compartments of broilers fed AA. Notably, the optimal dosage and duration of AA supplementation were crucial in influencing the observed effects on antioxidant capacity [14].

Inflammation and oxidative stress are linked. As AA levels in the diet increased from 0.3% to 0.6%, mRNA expression of IL-1β and TNF-α in the spleen of broilers decreased. The downregulation in the 0.6% AA group was most notable, suggesting superior anti-inflammatory action in these broilers. According to Hunt *et al*. [44] and Niu *et al*. [45], AA inhibits the generation of inflammatory cytokines. The expression of the anti-inflammatory IL-10 gene in the spleen was significantly increased in the groups receiving 0.6% and 0.9% AA supplementation compared to the control group (p ≤ 0.01). According to Fu *et al*. [46], AA can modify the immune response through the toll-like receptor 4/nuclear factor κB (NF-κB) signaling pathway. In addition, the anti-inflammatory and antioxidant potential of broilers was enhanced with dietary AA intake, as indicated by decreased mRNA expression of pro-inflammatory genes triggered by lipopolysaccharides [47–49]. The impact of *Artemisia* on TNF-α gene expression shows its anti-inflammatory potential in broilers [50]. Inhibiting the inflammatory cascade, AA modulates mitogen-activated protein kinase and decreases NF-κB activity [51].

The immunomodulatory function of AA in broilers is aided by its antioxidant and anti-inflammatory effects, derived from its flavonoid and phenolic content [9, 52, 53]. Li *et al*. [54] demonstrated that artemisinin extracted from AA suppresses IL-6 and IL-1β production, exhibiting anti-inflammatory attributes. TNF-α, a multitasking cytokine instrumental to host immunity and inflammatory responses in both acute and chronic stages, is identified as TNF-α. In addition, as reported by Song *et al*. [47], *Artemisia* was found to mitigate intestinal inflammation in heat-stressed broilers, leading to an increase in IL-10 production. IL-10, a potent and multifaceted anti-inflammatory cytokine, is renowned for its ability to inhibit the production of major pro-inflammatory cytokines. It also promotes upregulation of the humoral immune response and mitigates cell-mediated immune reactions, which are actions exerted by both innate and adaptive immune cells.

This study probed the economic consequences of AA supplementation for broilers. 0.6% AA intake resulted in the greatest breast return, giblet return, skeleton return, total return, and net return among the groups, including the control. 0.6% AA supplementation boosted the immune system, leading to the group’s economic advantage and profitability. AA supplementation results in increased profitability, immune system strengthening, and improved growth performance [11, 25].

## Conclusion

0.6% AA diet supplementation resulted in greater overall yield and profit than the control group. The addition of AA to the diet led to increased expression of CAT, SOD, and GSH-Px in the liver and spleen. Dietary AA upregulates IL-10, CAT, SOD, and GSH-Px mRNAs while downregulating IL-1β and TNF-α, ultimately reducing inflammation and enhancing immunological responses through antioxidant properties. AA, at an optimal dose of 0.6%, acts as a growth promoter and antibiotic alternative in chickens.

## Data Availability

The supplementary data can be available from the corresponding author on a request.

## Authors’ Contributions

MM, SHB, and SFS: Conceptualization and methodology. SFS, SHB, MMME, MM, TKE, AE, and DAA: Investigation and data curation and writing – original draft preparation and editing. All authors have read, reviewed, and approved the final manuscript.
